# Genetic variation of tandem repeats as a cause of Alzheimer’s Disease

**DOI:** 10.1002/alz.087066

**Published:** 2025-01-03

**Authors:** Alejandro Martin Trujillo, Bharati Jadhav, Paras Garg, Gabrielle Altman, Celine Manigbas, Mariya Shadrina, Andrew J Sharp

**Affiliations:** ^1^ Icahn School of Medicine at Mount Sinai Hospital, New York, NY USA

## Abstract

**Background:**

Alzheimer’s Disease (AD) is a common neurodegenerative disorder affecting >35 million people worldwide. Despite extensive genetic studies, the identified factors only explain a small fraction of the heritable risk of AD. This suggests the contribution of yet‐unknown genetic factors to the development of AD, such as tandem repeats (TRs). TRs are stretches of DNA consisting of two or more contiguous copies of a sequence of nucleotides (motif) arranged in a head‐to‐tail pattern (e.g., CAG‐CAG‐CAG). Recent studies indicate that rare expansions of short tandem repeats (STRs, motif size 1‐10 bp) as well as common copy number variation of variable number tandem repeats (VNTRs, motif size >10 bp) can act as risk modifiers for AD. However, despite this evidence, there has been no comprehensive evaluation of TR variation in large cohorts of AD patients. Therefore, we hypothesize that variation in TR sequences can contribute to the development of AD in a fraction of cases.

**Method:**

To uncover the link between TR variation and AD, we are performing a comprehensive profiling of TR variation using whole genome sequencing (WGS) data generated by the UK Biobank (https://www.ukbiobank.ac.uk/). This involves estimating copy numbers in ∼220,000 polymorphic loci, including 77,000 STRs and 150,000 VNTRs, across ∼349,000 genomes (∼9,000 cases and ∼340,000 unrelated controls) using novel bioinformatic approaches, followed by extensive quality control to ensure the reliability of our estimates. Subsequently, we will use the resulting high‐confident estimates to identify TR alleles that exhibit significantly different copy numbers in AD patients compared to controls.

**Result:**

To date, we have successfully designed efficient pipelines for profiling both STRs and VNTRs using WGS on a large scale to be executed on the UK Biobank cloud‐based environment (https://ukbiobank.dnanexus.com). Using these pipelines, we have generated copy number estimates in approximately 200,000 UK Biobank samples, including ∼1,200 AD cases.

**Conclusion:**

By leveraging the power of large cohorts and innovative methods, our study aims to explore the contribution of one of the most abundant and polymorphic, and yet understudied, genetic elements in the genome to AD, expanding our understanding of the genetic risk factors and mechanisms underlying this condition.

